# PaCO_2_ Association with Outcomes of Patients with Traumatic Brain Injury at High Altitude: A Prospective Single-Center Cohort Study

**DOI:** 10.1007/s12028-024-01982-8

**Published:** 2024-05-13

**Authors:** Eder Cáceres, Afshin A. Divani, Clio A. Rubinos, Juan Olivella-Gómez, André Emilio Viñan Garcés, Angélica González, Alexis Alvarado Arias, Kunal Bhatia, Uzma Samadani, Luis F. Reyes

**Affiliations:** 1 Unisabana Center for Translational Science, School of Medicine, Universidad de La Sabana, Chía, Colombia; 2 Department of Bioscience, School of Engineering, Universidad de La Sabana, Chía, Colombia; 3 Department of Critical Care, Clínica Universidad de La Sabana, Chía, Colombia; 4 Department of Neurology, The University of New Mexico, Albuquerque, NM USA; 5 Department of Neurology, University of North Carolina, Chapel Hill, NC USA; 6 Department of Neurology, University of Mississippi Medical Center, Jackson, MS USA; 7 Department of Neurosurgery, Minneapolis VA Health Care System, Minneapolis, MN USA; 8 Pandemic Sciences Institute, University of Oxford, Oxford, UK

**Keywords:** Traumatic brain injury, Neurocritical care, Carbon dioxide, Mechanical ventilation, Disability, Head injury, Trauma, Outcomes, High altitude

## Abstract

**Background:**

Partial pressure of carbon dioxide (PaCO_2_) is generally known to influence outcome in patients with traumatic brain injury (TBI) at normal altitudes. Less is known about specific relationships of PaCO_2_ levels and clinical outcomes at high altitudes.

**Methods:**

This is a prospective single-center cohort of consecutive patients with TBI admitted to a trauma center located at 2600 m above sea level. An unfavorable outcome was defined as a Glasgow Outcome Scale-Extended (GOSE) score < 4 at the 6-month follow-up.

**Results:**

We had a total of 81 patients with complete data, 80% (65/81) were men, and the median (interquartile range) age was 36 (25–50) years. Median Glasgow Coma Scale (GCS) score on admission was 9 (6–14); 49% (40/81) of patients had severe TBI (GCS 3–8), 32% (26/81) had moderate TBI (GCS 12–9), and 18% (15/81) had mild TBI (GCS 13–15). The median (interquartile range) Abbreviated Injury Score of the head (AISh) was 3 (2–4). The frequency of an unfavorable outcome (GOSE < 4) was 30% (25/81), the median GOSE was 4 (2–5), and the median 6-month mortality rate was 24% (20/81). Comparison between patients with favorable and unfavorable outcomes revealed that those with unfavorable outcome were older, (median age 49 [30–72] vs. 29 [22–41] years, *P* < 0.01), had lower admission GCS scores (6 [4–8] vs. 13 [8–15], *P* < 0.01), had higher AISh scores (4 [4–4] vs. 3 [2–4], *P* < 0.01), had higher Acute Physiology and Chronic Health disease Classification System II scores (17 [15–23] vs. 10 [6–14], *P* < 0.01), had higher Charlson scores (0 [0–2] vs. 0 [0–0], *P* < 0.01), and had higher PaCO_2_ levels (mean 35 ± 8 vs. 32 ± 6 mm Hg, *P* < 0.01). In a multivariate analysis, age (odds ratio [OR] 1.14, 95% confidence interval [CI] 1.1–1.30, *P* < 0.01), AISh (OR 4.7, 95% CI 1.55–21.0, *P* < 0.05), and PaCO_2_ levels (OR 1.23, 95% CI 1.10–1.53, *P* < 0.05) were significantly associated with the unfavorable outcomes. When applying the same analysis to the subgroup on mechanical ventilation, AISh (OR 5.4, 95% CI 1.61–28.5, *P* = 0.017) and PaCO_2_ levels (OR 1.36, 95% CI 1.13–1.78, *P* = 0.015) remained significantly associated with the unfavorable outcome.

**Conclusions:**

Higher PaCO_2_ levels are associated with an unfavorable outcome in ventilated patients with TBI. These results underscore the importance of PaCO_2_ levels in patients with TBI and whether it should be adjusted for populations living at higher altitudes.

**Supplementary Information:**

The online version contains supplementary material available at 10.1007/s12028-024-01982-8.

## Introduction

Traumatic brain injury (TBI) accounts for a substantial global health burden, with approximately 27 million cases reported annually, particularly in low-income and middle-income countries [1, 2]. As many as 50% of individuals with TBI do not regain their previous functionality [3], resulting in a reported age-standardized incidence rate of 111 (82–141) years lived with disability per 100,000 [1]. The most frequently cited factors related to poor outcomes include age, trauma severity, and the Glasgow Coma Scale (GCS) at presentation. Other factors, such as imaging findings, hypoxia, hypocapnia or hypercapnia, and hypotension, have also been identified [4–6]. These findings have allowed clinical teams and guidelines to establish goals in the acute setting to optimize care to limit secondary brain injury. These goals often include specific hemodynamic and respiratory parameters to achieve a particular target, such as optimal levels of partial pressure of carbon dioxide (PaCO_2_) [7, 8].

**Fig. 1:**
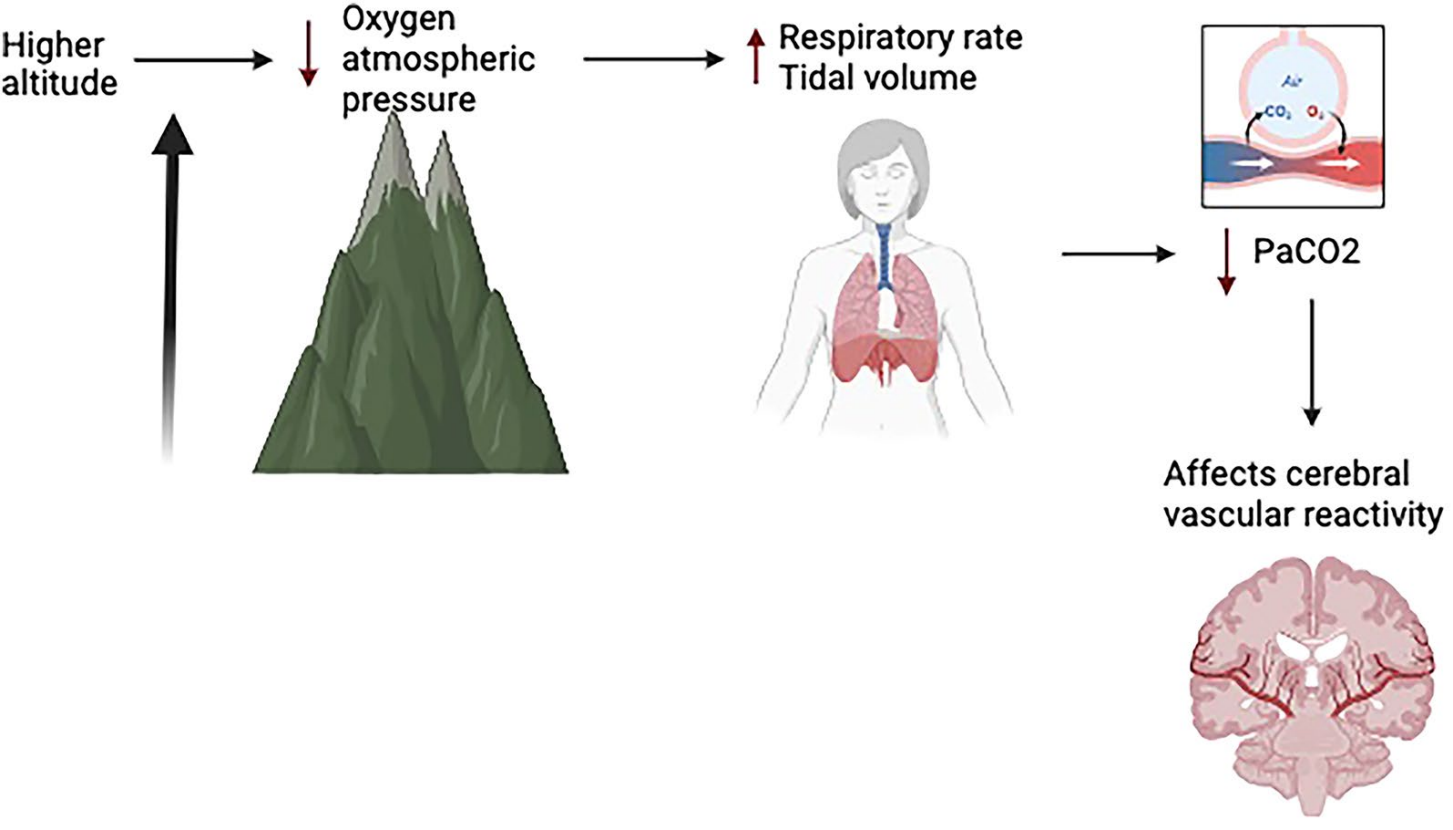
Effect of altitude on ventilation and cerebral vascular reactivity. Lower atmospheric pressure at a higher altitude leads to a compensatory increase in the minute ventilation, which reduces the PaCO_2_ level. How a lower baseline of PaCO_2_ affects the cerebral vasoreactivity, especially in TBI, and its therapeutic implications needs further investigation. PaCO_2_, partial pressure of carbon dioxide, TBI, traumatic brain injury

Carbon dioxide plays a central role in regulating cerebral blood flow, a notion supported by animal and human studies [9]. Hypercapnia causes blood vessels to dilate due to cerebrospinal fluid acidosis and the direct effect of extracellular H+ on vascular smooth muscle [10], whereas hypocapnia constricts them via alkalosis, influencing intracranial pressure and adjusting brain tissue perfusion in response to the environment [11]. Maintaining optimal PaCO_2_ levels is crucial in cases of brain injury because hypoperfusion and hypoxemia are closely linked to secondary brain injury and long-term consequences, impacting disability and survival rates [12, 13]. Guidelines recommend maintaining a target PaCO_2_ range between 35 and 45 mm Hg to prevent cerebral ischemia, in the case of low PaCO_2_ levels or hyperemia that could lead to elevated intracranial pressure if PaCO_2_ levels are high [6]. Several studies have reinforced this concept of targeting a specific range of PaCO_2_ as a goal of care for patients with TBI in the neurointensive care unit (neuro-ICU) [14] and its potential systemic implications [15, 16]. There is also considerable variability in the management of PaCO_2_ levels in patients with TBI within regions and centers [17]. Furthermore, evidence indicates that normal PaCO_2_ levels can vary according to altitude and barometric pressure [18, 19]. Generally, the barometric pressure is 760 mm Hg at sea level, with PaCO_2_ levels between 35 and 45 mm Hg being considered normal [19]. At higher altitudes, the atmospheric pressure of O_2_ and CO_2_ is lower, reducing PaO_2_ and PaCO_2_ (alveolar pressure), which in turn stimulates alveolar ventilation [20, 21]. The implications of these differences on the physiology and management of patients with TBI are unclear. Further contributions in this area may help guide the management and care of this patient population (Fig. 1).

We hypothesize that the TBI population at higher altitudes may benefit from different PaCO_2_ level targets compared with sea-level populations. Additionally, we hypothesized that the initial management of respiratory care and support in the acute phase might influence outcomes. This study evaluates the association between admission PaCO_2_ levels and outcomes at 6-month follow-ups in patients with TBI admitted to the neuro-ICU.

## Materials and Methods

The study was approved by the Institutional Review Board/Independent Ethics Committee under the local regulations and the Declaration of Helsinki for clinical practices, including obtaining informed consent from the patient representative. All clinical data were anonymized and collected using the Research Electronic Data Capture, an electronic data collection form provided by the Universidad de La Sabana.

### Study Population

This single-center prospective cohort study was conducted in a trauma center at the Universidad de La Sabana in Chía, Colombia. We consecutively recruited and collected data from patients with TBI admitted to the neuro-ICU from December 2019 to June 2022. The diagnosis, inclusion, and exclusion criteria, as well as imaging studies, were obtained by chart review.

The study cohort included ≥ 18-year-old patients with TBI admitted to the neuro-ICU within 24 h post injury and who stayed in the neuro-ICU for more than 48 h. Patients with a previous history of disability or debilitating diseases measured by a modified Rankin Scale > 2 and those admitted after 24 h post injury were excluded.

### Definitions

To evaluate the severity of TBI, we used the Abbreviated Injury Scale (AIS) of the head (AISh). We chose to use AISh because GCS was often obscured by sedation at the injury site or on admission to the neuro-ICU. AISh incorporates both clinical and imaging findings [22, 23], enabling a more nuanced assessment of the severity of the lesion (Table S1) and providing a robust correlation with outcomes. The AISh ranks injury on an ordinal scale 0 to 6 (from no injury to fatal). AISh can be classified as 1 (minor injury), 2 (moderate), 3 (serious), 4 (severe), 5 (critical), or 6 (fatal) [24, 25].

To assess the severity of the traumatic injury overall, the injury severity score (ISS) was used. The ISS is a composite measure derived from the AIS that includes a rating of the three most severely injured body regions and scores them on a range from 0 to 75. An ISS of 15 or higher is usually considered major trauma, and the compromise of two or more body regions with an AIS ≥ 3 is considered multiple traumas [25].

To characterize the severity of the brain injury in a head computed tomography (CT) scan, we used the Marshall classification. The Marshall scheme was first published in 1992 and uses six categories (I to VI) of increasing severity based on noncontrast CT scan findings, including midline shift, compression of cisterns, and mass lesions [26, 27] (Table S2). Its correlation with outcomes in TBI has been validated in several studies [28, 29].

The International Mission for Prognosis and Analysis of Clinical Trials (IMPACT) in TBI is a prognostic model that uses baseline characteristics and provides a probability of an unfavorable outcome and mortality at 6 months (Table S3). It defines an unfavorable outcome as a Glasgow Outcome Scale of 1–3. The IMPACT model has accurately discriminated outcomes after TBI [30, 31]. We used the laboratory model that includes age, motor score of the GCS, pupillary reactivity, CT characteristics, and information on admission hemoglobin and glucose levels [31].

To evaluate mortality and disability as outcomes, we selected the Glasgow Outcome Scale-Extended (GOSE), as outlined in Table S4, which is an ordinal scale of eight points ranging from death to good recovery [32]. GOSE has been used widely to assess outcomes in TBI [33–35]. A trained staff administered GOSE through a standardized phone interview with the patients or their caregivers 6 months post injury. For the analysis, we dichotomized GOSE into favorable and unfavorable outcomes. A favorable outcome (GOSE ≥ 4) was considered for those with upper severe disability to upper good recovery, and an unfavorable outcome was defined as a lower severe disability to death (GOSE < 4).

Infectious complications were evaluated using the Infectious Disease Society of America/American Thoracic Society guidelines definitions, including ventilator-associated pneumonia, ventilator-associated tracheitis, catheter-associated urinary tract infection, surgical site infection, and catheter-related bloodstream infection [36–39].

### Data Collection

Demographic data and trauma severity and prognostication scales that include the GCS, ISS, IMPACT model, and Marshall CT scan classification were recorded consecutively and prospectively. We collected vital signs and laboratory analysis from admission to the emergency department, which were reviewed and confirmed directly from the electronic medical record. Medical interventions during neuro-ICU stay, including mechanical ventilation, blood components transfusion, and use of vasopressors within 72 h of admission were reported. Finally, infections in the neuro-ICU, total hospital and neuro-ICU length of stay (LOS), and hospital mortality were also recorded. At the 6-month follow-up, patients or their legal representatives were contacted via phone by a trained research team member to administer the GOSE.

### Statistical Analysis

Continuous variables were summarized based on clinical relevance and distribution using minimum and maximum values, means ± standard deviations (SDs), or medians and interquartile ranges (IQRs). Dichotomous variables were presented as frequencies and percentages. Differences between intervention groups were assessed by applying the *χ*^2^ and Fisher’s exact tests for categorical variables. In contrast, continuous variables were evaluated using Student’s *t* test or the Mann–Whitney *U*-test, depending on their distribution.

A multivariate logistic regression model was constructed for the general cohort to investigate the risk factors associated with unfavorable outcomes at the 6-month follow-up. The model was adjusted for admission demographic data, vital signs, and laboratory tests. The logistic regression used the best subset method for the variable selection and included variables with a *P* value of less than 0.10 in the univariate analysis. Odds ratios (ORs) with 95% confidence intervals (CIs) were calculated based on the exponential values of the coefficients obtained from the final model D. We used R Studio (Version 2023.09.1+494) for the analysis.

## Results

### Patient Demographics and Characteristics

From December 2019 to June 2022, 81 patients with TBI admitted to the neuro-ICU at La Sabana Hospital in Chía, Colombia, located at 2600 m above sea level, were included in the study. The baseline and clinical characteristics are presented in Table 1. The median (IQR) age was 36 (25–50) years, and men accounted for 80% (*n* = 65) of the population. Traffic accidents were the leading cause of injury (60%, 49/81), followed by falls (24%, 19/81), cycling (9%, 8/81), violence (4%, 3/81), and others (3%, 2/81). Isolated TBI was present in 32% (26/81) of patients, and the most associated injuries were thorax 37% (30/81), limbs 20% (17/81), and abdomen 18% (14/81). The median (IQR) GCS on admission was 9 (6–14). The severity of TBI according to the AISh was moderate (AISh 2) in 27% (22/81) of patients, serious (AISh 3) in 27% (22/81) of patients, severe (AISh 4) in 33% (27/81) of patients, and critical (AISh 5) in 13% (10/81) of patients. The median (IQR) for AISh was 3 (2–4). In terms of the overall severity of trauma, the median (IQR) of ISS was 24 (13–32), among whom 72% (58/81) had major trauma (ISS > 15). Structural severity of head trauma was determined through the Marshall CT classification, in which 31% (24/81) of cases fell into diffuse injury I, 36% (31/81) fell into diffuse injury II, 8% (6/80) fell into diffuse injury III, 20% (16/81) fell into evacuated mass lesion V, 4% (3/81) fell into nonevacuated mass lesion VI, and 1% (1/81) fell into diffuse injury IV. The most frequent primary visible injury on head CT was contusion in 61% (50/81) of patients, followed by traumatic subarachnoid hemorrhage in 37% (30/81) of patients, subdural hematoma in 29% (24/81) of patients, epidural hematoma in 24% (20/81) of patients, and diffuse axonal injury in 11% (9/81) of patients. All patients were admitted to the neuro-ICU, and 60% (49/81) were placed on invasive mechanical ventilation. The neuro-ICU and hospital LOS were 6 (4–15) and 11 (6–23) days, respectively. Tracheostomy and gastrostomy were performed in 20% (16/81) and 15% (12/81) of the patients, respectively. The tracheostomy procedure was performed 10 (7–14) days post admission. At least one infectious complication was diagnosed in 30% (25/81) of patients during their neuro-ICU stay. Of the patients who had an infection, the sources of infection were ventilator-associated pneumonia in 28% (7/25), ventilator-associated tracheitis in 64% (16/25), catheter-associated urinary tract infection in 16% (4/25), one case of surgical site infection, and one case of sinusitis.

**Table 1:** Baseline characteristics of patients admitted to the neuro-ICU for TBI and comparison between groups with favorable and unfavorable outcomes

Characteristic	Overall (*n* = 81)	Favorable outcome (*n* = 56)	Unfavorable outcome (*n* = 25)	*P* value
Age, median (IQR)	36 (25–50)	29 (22–41)	49 (30–72)	< 0.01**
Sex male *n* (%)	65 (80)	34 (70)	23 (92)	0.18
Admission GCS, median (IQR)	9 (6–14)	13 (8–15)	6 (4–8)	< 0.01**
ISS, median (IQR)	24(13–32)	27 (20–34)	22 (12–29)	0.052
AIS head median (IQR)	3 (2–4)	3 (2–4)	4 (4–4)	< 0.01**
IMPACT-TBI outcome (%), median (IQR)	16 (7–38)	13 (6–17)	43 (31–64)	< 0.01**
Marshall Classification head CT *n* (%)	DI I: 24 (31)	DI I 22 (39)	DI I 2 (8)	0.009**
	DI II: 31 (36)	DI II 26 (46)	DI II 5 (20)	0.044*
	DI III: 6 (8)	DI III 2 (4)	DI III 4 (16)	0.1
	DI IV: 1 (1)	DI IV 0(0)	DI IV 1 (4)	0.6
	EML V: 16 (20)	EML V: 4 (7)	EML V 12 (48)	< 0.001**
	NEML VI: 3 (4)	NEML VI: 2 (3)	NEML 1 (4)	
APACHE II, median (IQR)	12 (7–17)	10 (6–14)	17 (15–23)	< 0.01**
Charlson score, median (IQR)	0 (0–0)	0 (0–0)	0 (0–2)	< 0.01**
Admission SBP, mean ± SD	122 ± 20	119 ± 19	128 ± 24	0.09
Admission Heart rate, mean ± SD	91 + 21	91 + 20	84 + 27	0.27
Admission Respiratory rate, median (IQR)	20 (18–22)	19 (18–21)	20 (18–22)	0.3
Admission WBC × 10^3^/dl mean ± SD	16 ± 5	16,9 ± 4	15.1 (5,4)	0.16
Admission Hemoglobin, median (IQR), grs/dl	14(12–15)	14,2 (12,7–14,9)	14 (12–15)	0.4
Admission Platelets, mean ± SD	231 ± 79	240 ± 88	213 ± 66	0.15
Admission Serum sodium, median (IQR), mEq/L	139 (137–142)	140 (138–141)	139 (137–142)	0.38
Admission PaO_2_, median (IQR), mmHg	85 (71–123)	86 (70–127)	88 (72–124)	0.7
Admission PaCO_2_ mmHg, mean ± SD	35 ± 8	32 ± 6	39 ± 9	< 0.01**
Admission serum lactate, median (IQR), mmol/L	2.7 (1,8–3,9)	2.7(1,9–3,8)	2.8 (1,8–4,3)	0.6
Serum glucose, median (IQR), mg/dl	130 (120–160)	130 (120–160)	140 (120–160)	0.3
Mechanical ventilation *n* (%)	49 (60)	27 (49)	22 (88)	0.003**
Days on mechanical ventilation, median (IQR)	6 (3–12)	3 (2–8)	10 (4–18)	0.008**
Vasopressors within 72 h *n* (%)	45 (55)	24 (44)	21 (84)	0.002**
Neurosurgical intervention n (%)	20 (24%)	5 (9)	15 (60)	< 0.01**
Infectious complication *n* (%)	25 (30)	9 (20)	14 (56)	0.004**
Tracheostomy *n* (%)	16 (20)	2 (4)	12 (48)	< 0.01**
Neuro-ICU LOS, median (IQR), days	6 (4–15)	5 (4–8)	14 (6–23)	0.002**
Hospital LOS, median (IQR), days	11 (6–23)	11 (6 – 13)	22 (7—43)	0.06

*AIS head* abbreviated injury score of the head, *DI I* diffuse injury I, *DI II* diffuse injury II, *DI III* diffuse injury III, *DI IV* diffuse injury IV, *EML V* evacuated mass lesion V, *GCS* Glasgow Coma Scale, *IMPACT TBI* International Mission for Prognosis and Analysis of Clinical Trials in Traumatic Brain Injury, *ISS* Injury Severity Score, *NEML VI* Non.-evacuated Mass Lesion VI, *neuro-ICU* neurointensive care unit, *SBP* systolic blood pressure, *WBC* white blood cell count. *P < 0.05, **P < 0.01

### Outcome

At 6 months post injury, we were able to conduct phone interviews with all survivors or their caregivers to administer the GOSE (*n* = 71). Patients who died during the hospitalization (*n* = 10, 12%) were included in the unfavorable outcome group. A total of 56 patients had a favorable outcome (GOSE 4–8) and 25 patients had an unfavorable outcome (GOSE 1–3). The frequency of an unfavorable outcome (GOSE < 4) was 30% (25/81) at 6 months. The median (IQR) GOSE at 6 months was 4 (2–5). Mortality at 6 months was 24% (20/81). When applying the IMPACT laboratory model to the entire cohort, the median (IQR) probability of a 6-month unfavorable outcome was 16% (7–38%).

**Fig. 2:**
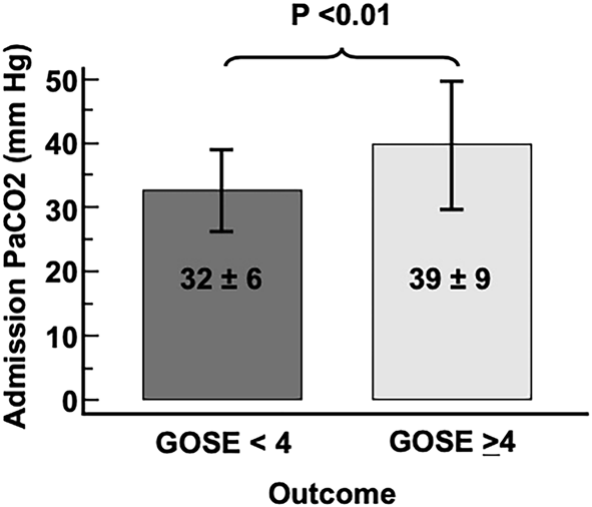
Admission PaCO_2_ levels (mean ± SD) for the 6-month outcome for patients with TBI. Glasgow Outcome Scale-Extended (GOSE) scores for unfavorable outcomes (< 4) and favorable outcomes (≥ 4). *PaCO*_*2*_ partial pressure of carbon dioxide, *SD* standard deviation, *TBI* traumatic brain injury

Comparison between patients with a 6-month favorable outcome and unfavorable outcome (Table 1) revealed that those with an unfavorable outcome were older (49 [30–72] vs. 29 [22–41] years, *P* < 0.01) and had lower admission GCS scores (6 [4–8] vs. 13 [8–15], *P* < 0.01), higher AISh (4 [4–4] vs. 3 [2–4], *P* < 0.01), increased probabilities of poor outcome by the IMPACT model (%) (43 [31–64] vs. 13 [6–17], *P* < 0.01), higher APACHE II (Acute Physiology and Chronic Health Evaluation II) scores (17 [15–23] vs. 10 [6–14], *P* < 0.01), higher Charlson scores (0 [0–2] vs. 0 [0–0], *P* < 0.01), and higher PaCO_2_ levels (39 ± 9 vs. 32 ± 6 mm Hg, *P* < 0.01; Fig. 2). In terms of hospital variables and interventions, the group with an unfavorable 6-month outcome was more frequently on mechanical ventilation (88% (22/25) vs. 41% (27/56), *P* < 0.01) and it required vasopressors in 84% (21/25) versus 48% (24/56) of patients, *P* < 0.01. The group with the unfavorable outcomes also required neurosurgical intervention, 60% (15/25) versus 9% (5/56), and it underwent tracheostomy in a greater proportion during neuro-ICU stay (48% [12/25] vs. 4% [2/56]). Other data collected on admission include systolic blood pressure, heart rate, respiratory rate, white blood cell count, platelet count, serum sodium, lactate, PaO_2_, hemoglobin, and serum glucose; none of these variables were different between the groups with favorable and unfavorable outcomes (Table 1).

### PaCO_2_ and Outcome for Patients on Mechanical Ventilation

**Fig. 3:**
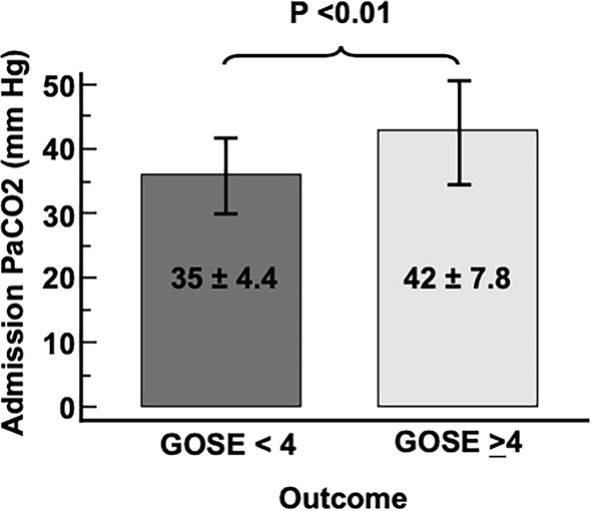
Admission PaCO_2_ levels (mean ± SD) for the 6-month outcome for patients with TBI admitted to the neuro-ICU on mechanical ventilation. Glasgow Outcome Scale-Extended (GOSE) for unfavorable outcomes (< 4) and favorable outcomes (≥ 4). *neuro-ICU* neurointensive care unit, *PaCO*_*2*_ partial pressure of carbon dioxide, *SD* standard deviation, *TBI* traumatic brain injury

When evaluating the group on mechanical ventilation (*n* = 49), the PaCO_2_ mean ± SD was 39.0 ± 7.7 mm Hg, which was significantly higher for those with a 6-month unfavorable outcome compared with the group with a favorable outcome (42.0 ± 7.8 vs. 35.3 ± 4.4, *P* < 0.01; Fig. 3). In the group without ventilatory support, the PaCO_2_ mean ± SD was 28.1 ± 5.8 mm Hg, and it was significantly lower for the group with an unfavorable outcome (21.6 ± 2.5 vs. 28.9 ± 5.6, *P* < 0.01) compared with those with favorable outcome at 6 months. Mean PaCO_2_ levels were lower in the group without ventilator support than in those on mechanical ventilation (28.1 ± 5.8 vs. 39.0 ± 7.7, *P* < 0.001). Finally, neuro-ICU LOS was longer for the unfavorable outcome group, 14 (6–23) versus 5 days (4–8), *P* < 0.01.

### Logistic Regression Analysis

Univariate analysis of in-hospital variables and their association with a 6-month unfavorable outcome were performed through a univariate logistic regression (*P* < 0.1) (Table 2). Variables significantly associated with the primary outcome included age (OR 1.01, 95% CI 1.01–1.02), GCS (OR 1.60, 95% CI 1.30–2.10), AISh (OR 1.21, 95% CI 1.11–1.32), use of vasopressors within 72 h of admission (OR 1.5, 95% CI 1.20–1.80), mechanical ventilation (OR 1.4, 95% CI 1.16–1.8), infectious complications (OR 1.45, 95% CI 1.2–1.8), neurosurgical intervention (OR 1.6, 95% CI 1.3–2.0), and need for a tracheostomy (OR 1.8, 95% CI 1.4–2.3). Regarding the laboratory data on admission, the one with a significant association was PaCO_2_ (OR 1.02, 95% CI 1.01–1.03). From the variables with a significant association in the univariate analysis (*P* < 0.1), age, AISh, APACHE II, and PaCO_2_ levels were included in the multivariate analysis. From those, age (OR 1.14, 95% CI 1.1–1.30, *P* < 0.01), AISh (OR 4.7, 95% CI 1.55–21.0, *P* < 0.05), and PaCO_2_ levels (OR 1.23, 95% CI 1.10–1.53, *P* < 0.05) remained significantly associated with the 6-month unfavorable outcome in the multivariate analysis (Table 2).

**Table 2:** Univariate and multivariate analysis of variables on admission and a 6-month unfavorable outcome in patients with TBI admitted to the neuro-ICU

Variables	Univariate Analysis		Multivariate analysis	
	OR (95% CI)	*P* value	OR (95% CI)	*P* value
Demographics				
Age	1.01 (1.01–1.02)	< 0.01**	1.14 (1.10–1.30)	< 0.01**
Severity of injury				
Admission GCS	1.60 (1.30–2.10)	< 0.01**		
AIS head	1.21 (1.11–1.32)	< 0.01**	4.7 (1.55–21.0)	0.016*
APACHE II	1.04 (1.03–1.05)	< 0.01**	0.90 (0.70–1.30)	0.90
Medical interventions				
Vasopressor requirement^a^	1.50 (1.20–1.80)	< 0.01**		
Transfusion of blood components^a^	1.20 (0.90–1.50)	0.14		
Invasive mechanical ventilation	1.40 (1.16–1.80)	< 0.01**		
Labs on admission				
Leucocytes, cell/mm10^3^	0.98 (0.95–1.01)	0.13		
Hemoglobin, g/dL	0.97 (0.92–1.02)	0.30		
Platelets, cell/mm^3^	0.99 (0.99–1.00)	0.18		
Serum sodium, mEq/L	0.99 (0.97–1.00)	0.90		
PaO_2_, mmHg	1.00 (0.99–1.00)	0.80		
PaCO_2_, mmHg	1.02 (1.01–1.03)	< 0.01**	1.23 (1.10–1.53)	0.026*
Lactic acid, mmol/L	1.00 (0.95–1.03)	0.50		
Glucose, mgr/dL	1.00 (0.99–1.00)	0.19		
BUN	1.00 (0.97–1.02)	0.80		
Creatinine	1.20 (0.80–1.70)	0.20		

*GCS* Glasgow Coma Scale, *AIS* abbreviated injury score, *BUN* blood ureic nitrogen

^a^Within 72 h of admission; *P < 0.05,   **P < 0.01

Afterward, the same analysis was applied to the subgroups of patients with and without ventilator support. A multivariate analysis was performed on the mechanical ventilation group using the same variables: age, AISh, APACHE II, and PaCO_2_ levels. In this case, again, AISh (OR 5.4, 95% CI 1.61–28.5, *P* = 0.017) and PaCO_2_ levels (OR 1.36, 95% CI 1.13–1.78, *P* = 0.015) remained significantly associated with the 6-month unfavorable outcome (Table 3). The same analysis for the group without mechanical ventilation did not yield a significant result for any of the variables (*P* = 0.99). The Hosmer–Lemeshow test for binary logistic regression models demonstrated the goodness-of-fit test (*P* = 0.97).

**Table 3:** Multivariate analysis of clinical variables and a 6-month unfavorable outcome in TBI patients admitted to the neuro-ICU on mechanical ventilation

Variables	Multivariate analysis	
	OR (96% CI)	*P* value
Age	1.09 (1.01–1.24)	0.06
Apache II	1.2 (0.98–1.59)	0.10
AIS head	5.4 (1.61–28.5)	0.017*
PaCO_2_ on admission	1.36 (1.13–1.78)	0.015*

*AIS* abbreviated injury score; *P < 0.05

## Discussion

This study initially characterizes a prospective cohort of patients with TBI admitted to the neuro-ICU in an academic center in the Andean region in Colombia. The group with an unfavorable outcome was older and had lower GCS scores on admission, higher AISh, higher probabilities of an unfavorable outcome by the IMPACT TBI model, higher APACHE II, and higher Charlson scores. Among vital signs and laboratory data, the only documented difference was a higher PaCO_2_ levels on admission for those with an unfavorable outcome. In terms of in-hospital procedures, the group with an unfavorable outcome required more ventilatory and hemodynamic support, underwent neurosurgical interventions and tracheostomy more often, and had a longer LOS in the neuro-ICU. After adjusting for age, severity of TBI, and APACHE II, PaCO_2_ levels remained directly correlated with an unfavorable outcome at 6 months. A higher PaCO_2_ level was associated with an unfavorable 6-month outcome for all the study groups and the group on ventilatory support. In the subgroup, without ventilatory support, this correlation was not maintained. The mean PaCO_2_ level in the subgroup without ventilatory support was lower than those on mechanical ventilation. The lower PaCO_2_ levels observed in the nonventilated group may be associated with the inherently lower baseline levels of PaCO_2_ in populations residing at higher altitudes. Consequently, this suggests a potential difference in the way regulatory mechanisms are established [9, 12].

The demographic characteristics of the studied cohort are similar to what others have found in terms of age and cause of trauma [40, 41]. TBI affects predominantly the adult male population in their fourth or fifth decade of life, and the leading causes of injury are road accidents and falls. This has been consistent in several prospective studies, including the European and Chinese cohorts of CENTER-TBI (Collaborative European NeuroTrauma Effectiveness Research in Traumatic Brain Injury) and the TRACK-TBI (Transforming Research and Clinical Knowledge in Traumatic Brain Injury ) for the United States [4, 40, 42]. Regarding mortality and functional outcomes, the ICU stratum of the European Center-TBI found 43.1% and 21.3% rates of an unfavorable outcome (GOSE < 5) and mortality, respectively. The results in our study are similar in both mortality (24%) and unfavorable outcome (30%), bearing in mind that the definition we used for unfavorable outcome was GOSE < 4 [42]. There is no standardized manner to dichotomize GOSE, and definitions vary across studies [43, 44]. Patients with TBI might show functional and cognitive improvement even 1 year after the trauma [45, 46], depending on their recovery trajectory. A GOSE score equal to 4 refers to a person who requires partial supervision and assistance but can be on their own at home for at least 8 h a day. Therefore, we considered it reasonable to define GOSE ≥ 4 as the favorable outcome, considering that those patients are already partially independent at home and still have the potential for further progress.

Several studies have pointed out that older and more severely injured patients with TBI have more frequent severe disability and functional dependence after TBI [47, 48]. Patients with moderate and severe TBI are usually admitted to the neuro-ICU, where interventions are guided by targets that aim to protect the brain from a secondary injury [49]. Henceforth, it is also the patient with more severe trauma who needs more assistance in terms of respiratory, hemodynamic, and metabolic support as well as surgical interventions [50, 51]. In our cohort, the group with unfavorable outcomes was older and had a more severe TBI on admission. Therefore, it could be expected that it is, in turn, the group that received a higher burden of care, including mechanical ventilation, vasopressors, neurosurgical, and tracheostomy procedures, and that was more exposed to complications, such as in-hospital infections and longer ICU stays. This reflects the complexity of treatment and prognosis when many factors are involved, leaving aside the variability of management across centers and regions [51]. Despite this challenge, some prognostic models have been developed and validated, for instance, the Corticosteroid Randomization After Significant Head Injury model and the International Mission for Prognosis and Analysis of Clinical Trials (IMPACT) in TBI model [52–54]. These models estimate the probability of disability and mortality and consider factors such as age, Glasgow motor score, pupillary reactivity, and imaging findings on head CT scans. We did not intend to develop a model, but we did identify some factors on admission associated with outcomes, including age, severity of TBI, APACHE II, and the need for hemodynamic and ventilatory support. However, when assessing vital signs and laboratory tests, higher levels of PaCO_2_ on admission were associated with the unfavorable outcome, even after controlling for the age and severity of the injury. The role of PaCO_2_ in this context relies on its effect on the cerebral vasculature or vasoreactivity [55, 56]. The brain has high metabolic demand, requiring a constant supply of oxygen and glucose [57]. This supply is ensured through a tightly regulated cerebral blood flow that matches each brain region’s temporal and spatial metabolic requirements [58]. One of those mechanisms is the vasomotor response to carbon dioxide, in which cerebral arterioles dilate or contract according to changes in PaCO_2_ levels. This response has a sigmoidal shape and functions within the 20–60 mm Hg of PaCO_2_. Every 1–mm Hg increase in PaCO_2_ corresponds to roughly a 4% increase in cerebral blood flow [59, 60], which in turn increases the cerebral blood volume, resulting in an intracranial pressure elevation and finally affecting the cerebral perfusion pressure. Several cohorts have demonstrated the effect of PaCO_2_ management on outcomes, including mortality [19]. However, variability in management exists across centers [61]. Guidelines recommend a normal range ventilation, PaCO_2_ levels 35–45 mm Hg, and avoidance of hyperventilation and severe (< 25 mm Hg) or moderate (< 30 mm Hg) hypocapnia [7, 8] given the risk of brain ischemia.

In our cohort, we found higher PaCO_2_ levels for those patients with an unfavorable outcome, and the multivariate analysis revealed a direct relation between admission PaCO_2_ levels and the probability of death and disability. The association remained for the subgroup on mechanical ventilation but not for those patients without ventilatory support. This could be expected, given that PaCO_2_ levels in a ventilated patient depends mostly on the ventilator settings and can be adjusted to a specific goal. However, we would like to point out that most of our patients had PaCO_2_ levels within the recommended range of 35–45 mm Hg and even below for those with a favorable outcome, 32 ± 6 mm Hg. In addition, nonventilated patients had even lower PaCO_2_ levels. These results underscore the importance and impact of PaCO_2_ as a crucial target in the management of ventilated patients with TBI and raise the question of whether, for populations at higher altitudes, different PaCO_2_ goals should be pursued. Further investigation would be needed to answer this question, which will benefit a substantial proportion of the global TBI population living at higher altitudes.

A limitation of our study includes being a single-center study that requires further validation to make the results more generalizable. In addition, we only recorded the admission PaCO_2_ values rather than serial values.

## Conclusions

We evaluated the relationship between PaCO_2_ levels and functional outcomes in patients with TBI admitted to the neuro-ICU. Interestingly, in our center, situated at a higher altitude above sea level, we observed that in the sample of patients on mechanical ventilation, a PaCO_2_ below the recommended target was associated with improved outcomes. Although this is a single-center prospective cohort study, it raises the question of whether the target PaCO_2_ levels need adjustment in populations at higher altitudes.

## Supplementary Information

Below is the link to the electronic supplementary material.

Supplementary file1 (DOCX 21 KB)
