# Supplementary material for: PaCO2 Association with Outcomes of Patients with Traumatic Brain Injury at High Altitude: A Prospective Single-Center Cohort Study
Source: Neurocrit Care. 2024 May 13;41(3):767–78. doi: 10.1007/s12028-024-01982-8 (PMC11599390; doi:10.1007/s12028-024-01982-8)
Supplement: Supplementary file 1 — Supplementary file1 (DOCX 21 KB) [file 12028_2024_1982_MOESM1_ESM.docx]

**Table S1**. Abbreviated Injury Scale of the Head (24)

| AIS score | 1. Minor | 1. Moderate | 1. Severe not life-threatening | 1. Severe life-threatening | 1. Critical, survival uncertain |
| --- | --- | --- | --- | --- | --- |
| **Head** | **Awake on admission or initial observation:**  No prior unconsciousness but may have headache/dizziness 2o to head trauma | **Awake on admission or initial observation:**  Prior unconsciousness but length of time unspecified.  Amnesia (no recollection of crash).  Unconsciousness < 15 min.  **Fracture of vault** (frontal, occipital, parietal, sphenoid, temporal or unspecified, closed, undisplaced, diastatic, linear, simple. | **Awake on admission or initial observation:**  Prior unconsciousness but length unspecified/amnesia.  Unconsciousness 15 min with neurological deficit.  Unconsciousness 15-59 min.  **Lethargic, stuporous, obtunded on admission or initial observation** (can be aroused by verbal stimuli.  **Unconsciousness on admission or initial observation** (unresponsive to verbal commands) < 1 h.  **When level of consciousness on admission or initial observation is unknown:**  Unconsciousness 15-59 min. Unconsciousness < 15 min with neurological deficit.  **Fracture of base** (basilar, ethmoid, orbital roof, sphenoid, temporal) without CSF leak *.  Comminuted compound, depressed or displaced fracture of vault.  **Cerebellum, cerebrum:**  Contusion, injury involving any of the following but not further anatomic description (subarachnoid hemorrhage, edema, brain swelling, subpial hemorrhage, hygroma, ischemia, infarction). | **Awake on admission or initial observation:**  Unconscious 15-59 min with neurological deficit.  Lethargic, stuporous, obtunded on admission or initial observation (can be aroused by verbal stimuli). Prior unconsciousness for unspecified length of time involving neurological deficit.  **Unresponsive on admission or initial observation** (unresponsive to verbal commands):  1-24 h (includes 1 calendar day when hours cannot be estimated). Appropriate movements but only upon painful stimuli (no matter the length of unconsciousness). Length of unconsciousness unspecified/unconscious < 1 h involving neurological deficit.  **When level of unconsciousness on admission or initial observation is unknown, but unconscious for:**  1-24 h (includes 1 calendar day when hours cannot be estimated). 15-59 min involving neurological deficit.  **Fracture of base** (basilar ethmoid, orbital roof, sphenoid, temporal with CSF leak * /pneumocephalus).  **Fracture of vault** (frontal, occipital, parietal, sphenoid, temporal, unspecified) open, dura torn, CSF leak, pneumocephalus or brain exposed.  **Cerebellum or cerebrum** laceration hematoma epidural/subdural < 100 cc or unspecified, hematoma intracerebral, intracerebellar (including petechial and subcortical hematoma) | **Unconscious on admission or initial observation** (unresponsive to verbal stimuli):  Inappropriate movements (decerebrate, decorticate, flaccid, no response to pain- no matter the length of unconsciousness.  1-24 h (includes 1 calendar day when hrs cannot be estimated) / appropriate movements but only upon painful stimuli (no matter the length of unconsciousness with neurological deficit.  **When level of consciousness on admission or initial observation is unknown but unconscious for**: 1-24 h with neurological deficit. > 24 hrs.  **Brain stem:** compression, contusion, injury involving hemorrhage.  **Cerebellum or cerebrum:** hematoma, epidural, subdural > 100 cc diffuse brain injury / white matter shearing injury. |

* CSF: cerebrospinal fluid

**Table S2**. Marshall classification on head CT scan (28)

| **Classification** | **Definition** |
| --- | --- |
| Diffuse injury I | No visible intracranial pathology. |
| Diffuse injury II | Cisterns present, midline shift ≤5 mm and/or lesion densities, no high/mixed density lesion >25 cm³. |
| Diffuse injury III | Cisterns compressed or absent, midline shift ≤5 mm, no high/mixed density lesion >25 cm³. |
| Diffuse injury IV | Midline shift >5 mm, no high/mixed density lesion >25 cm³. |
| Evacuated lesion V | Any surgically-evacuated lesion. |
| Non-evacuated lesion VI | High/mixed density lesion >25 cm³, not surgically evacuated. |

**Table S3**. IMPACT TBI model (33)

| **CORE MODEL** | |
| --- | --- |
| **Variable** |  |
| Age | Years |
| Motor score (if on sedation/paralysis or unable to be assessed mark as “Not testable”. | Obeys commands  Localizes to pain  Withdrawal from pain  Flexion to pain  Extension to pain  No motor response  Not testable |
| Pupils | Both reactive  One reactive  Neither reactive |
| **EXTENDED MODEL**  **Core model variables in addition to:** | |
| Marshall CT classification | Diffuse injury I  Diffuse injury II  Diffuse injury III or IV  V o VI |
| Traumatic subarachnoid hemorrhage on CT | Yes or No |
| Epidural hematoma on CT | Yes or No |
| Hypoxia | Yes, or suspected  No |
| Hypotension | Yes, or suspected  No |
| **LAB MODEL**  **Core and extended model variables in addition to:** | |
| Glucose | mg/dl |
| Hemoglobin | gr/dl |

**Table S4.** Glasgow Outcome Scale-Extended (GOSE) (34)

| 1. Dead |  |
| --- | --- |
| 1. Vegetative state | Condition of unawareness with only reflex responses but with periods of spontaneous eye opening. |
| 1. Lower severe disability | Patient full dependent for all activities of daily living. Requires assistance to be available constantly. Unable to be left alone at night. |
| 1. Upper severe disability | Can be left at home for up to eight hours but remains dependent. Unable to use public transport or shop by themselves. |
| 1. Lower moderate disability | Able to return to work but in sheltered workshop or non-competitive job. Rarely participates in social and leisure activities. Ongoing daily psychological problems (anxiety, mood swings, depression, tempers). |
| 1. Upper moderate disability | Able to return to work but a reduced capacity. Participates in social and leisure activities less than half as often. Weekly psychological problems. |
| 1. Lower good recovery | Return to work. Participates in social and leisure activities a little less and has occasional psychological problems. |
| 1. Upper good recovery | Full recovery with no current problems related to the injury. |
